# Engineering the band gap and optical properties of a two-dimensional molybdenum carbon fluoride MXene

**DOI:** 10.1107/S2052520622007387

**Published:** 2022-08-16

**Authors:** Doan Thi Kieu Anh, Luong Viet Mui, Pham Hong Minh, Nguyen Thanh Binh, Marilou Cadatal-Raduban

**Affiliations:** a Institute of Physics, Vietnam Academy of Science and Technology, 10 Dao Tan, Ba Dinh, Hanoi, Vietnam; b Osaka University ASEAN Campus Vietnam, 18 Hoang Quoc Viet, Cau Giay, Hanoi, Vietnam; c Graduate School of Engineering, Osaka University, 2-1 Yamadaoka, Suita, Osaka 565-0871, Japan; d Centre for Theoretical Chemistry and Physics, School of Natural Sciences, Massey University, Auckland 0632, New Zealand; e Institute of Laser Engineering, Osaka University, 2-6 Yamadaoka, Suita, Osaka 565-0871, Japan; University of Geneva, Switzerland

**Keywords:** two-dimensional materials, MXene, monolayer, multilayer, band gap engineering, electronic properties, optical properties, crystal structure

## Abstract

Using first-principles density functional theory, we investigated the electronic and optical properties of monolayer and multilayer nanosheets of molybdenum carbon fluoride (Mo_2_CF_2_), a two-dimensional (2D) transition-metal carbide MXene. The indirect band gap of the Mo_2_CF_2_ semiconductor can be engineered by controlling the number of layers where the band gap energy changes from 0.278 eV for the monolayer to 0.249 eV for the trilayer nanosheet. The decrease in band gap energy in the multilayer is due to inter­layer coupling, which splits the bands according to the number of layers. Mo_2_CF_2_ behaves as a metal with an anomalous dispersion and high optical conductivity at incident photon energies of 0.68–2.19, 3.49–6.68 and 7.30–8.31 eV. It has a relatively low reflectivity and is absorbing over a broad range of photon energies from about 0.429 (2890), 0.387 (3204) and 0.345 eV (3594 nm) for the monolayer, bilayer and trilayer nanosheets, respectively, achieving peak absorption in the vacuum ultraviolet region at about 7.9 eV (157 nm). The optical properties of Mo_2_CF_2_ can likewise be tuned by varying the number of layers. The unique behavior of its optical properties along with the ability to control its electronic and optical properties enhances the potential of 2D Mo_2_CF_2_ for various applications in the fields of electronics and energy storage.

## Abstract 2

Using first-principles density functional theory, the electronic and optical properties of monolayer and multilayer nanosheets of molybdenum carbon fluoride (Mo_2_CF_2_), a two-dimensional (2D) transition-metal carbide MXene, were investigated. The unique behavior of its optical properties along with the ability to control its electronic and optical properties enhances the potential of 2D Mo_2_CF_2_ for various applications in the fields of electronics and energy storage.

## 1. Introduction

Since the discovery of graphene and its wonderful properties (Kaushik *et al.*, 2019 ▸; Ostovari *et al.*, 2018 ▸), two-dimensional (2D) materials with their extraordinary electronic, mechanical and optical properties (Georgiou *et al.*, 2012 ▸; Tamleh *et al.*, 2018 ▸) have attracted much research inter­est in the field of materials science. The recent discovery of a novel family of com­pounds called 2D transition-metal carbides, nitrides and carbonitrides (MXene) has gained significant attention from the scientific community and has spurred new inter­est in 2D materials (Khazaei *et al.*, 2019 ▸; Bae *et al.*, 2021 ▸; Naguib *et al.*, 2013 ▸; Anasori *et al.*, 2015 ▸). MXene has a general formula of *M_n_*_+1_ *X_n_T_x_* (*n* = 1–3), where *M* indicates early transition metals (*e.g.* Sc, Ti, V, Nb, Mo, *etc*.), *X* stands for C and/or N, and *T_x_* represents functional groups on the surface of the MXene, such as –OH, –O or –F (Naguib *et al.*, 2011 ▸; Hong *et al.*, 2020 ▸; Champagne *et al.*, 2018 ▸). MXenes have a huge potential in a wide range of applications, such as energy storage (Yorulmaz *et al.*, 2016 ▸; Anasori *et al.*, 2017 ▸), sensing (Sinha *et al.*, 2018 ▸; Khazaei *et al.*, 2012 ▸), catalysis (Peng *et al.*, 2018 ▸; Wang *et al.*, 2018 ▸; Li & Wu, 2019 ▸) and the development of electronic devices (Zhang & Nicolosi, 2019 ▸).

Molybdenum carbon fluoride (Mo_2_CF_2_) is a MXene consisting of the molybdenum (Mo) transition metal, carbon (C) and functionalized by fluorine (F). Khazaei *et al.* (2014 ▸) reported that a monolayer Mo_2_CF_2_ is an indirect band gap semiconductor with a narrow band gap of 0.27 eV and the most stable Mo_2_CF_2_ (hollow site–hollow site) structure is a promising thermoelectric material. Monolayer Mo_2_CF_2_ has many excellent properties, such as high capacity, outstanding mechanical strength and good flexibility. Therefore, this material has a great potential as an anode material for Li-ion batteries, as well as in various applications, including electronics and energy storage (Mehta *et al.*, 2019 ▸).

Despite previous works on several MXenes, including Ti_3_C_2_T_2_, Nb_4_C_3_ *T_x_* and V_2_C– , with a wide variety of applications (Khazaei *et al.*, 2019 ▸; Bae *et al.*, 2021 ▸; Champagne *et al.*, 2018 ▸; Mostafaei & Abbasnejad, 2021 ▸; Khan *et al.*, 2019 ▸), research on the electronic and optical properties of Mo_2_CF_2_, and how the number of layers affects these properties is still lacking. The ability to control the band gap of a material is very important for its application in the field of electronic devices. As a material with exciting prospects for electronics and energy storage applications, the ability to control the electronic structure and hence the band gap of Mo_2_CF_2_ will greatly enhance its potential. However, the ability to control the band gap of Mo_2_CF_2_ has not yet been demonstrated. Therefore, this work investigates the structural, electronic and optical properties of monolayer, bilayer and trilayer nanosheets of Mo_2_CF_2_ (collectively referred to as 2D layered Mo_2_CF_2_) using first-principles calculations in the framework of the generalized gradient approximation (GGA) implemented by the Perdew–Burke–Ernzerhof (PBE) functional. Our results illustrate how the electronic structure, band gap energy and optical properties can be controlled by manipulating the number of layers in a 2D Mo_2_CF_2_.

## 2. Computational method

**Figure 1:**
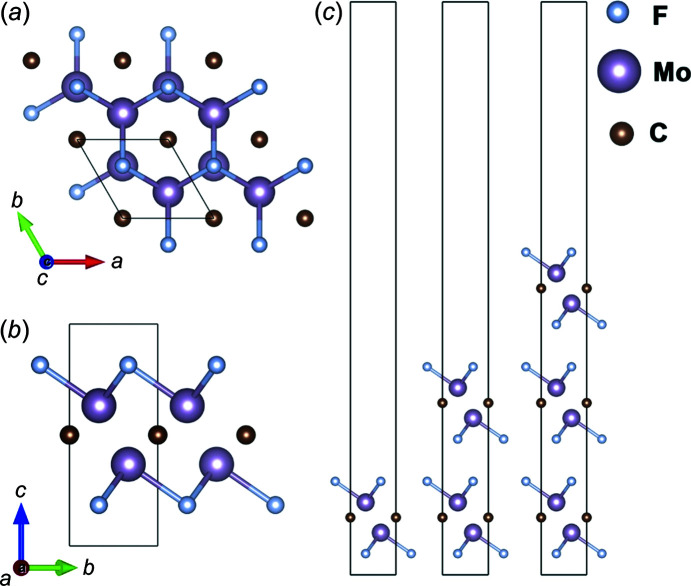
The crystal structures of Mo_2_CF_2_ showing (*a*) a top view and (*b*) a side view of bulk Mo_2_CF_2_, and (*c*) a side view of the monolayer (left), bilayer (middle) and trilayer (right) nanosheets of Mo_2_CF_2_.

The crystal structures of the bulk and 2D layered Mo_2_CF_2_ were visualized using *VESTA* (Momma & Izumi, 2011 ▸). The top and side views of bulk Mo_2_CF_2_ are shown in Figs. 1 ▸(*a*) and 1 ▸(*b*). Bulk Mo_2_CF_2_ consists of a layer of C atoms sandwiched between Mo atoms and two layers of Mo atoms halogenated by F atomic layers on each side. It has a hexa­gonal structure and space group *P*6_3_/*mmc* (No. 194). The side view of the relaxed configurations of the monolayer, bilayer and trilayer nanosheet is illustrated in Fig. 1 ▸. The atomic coordinates used to construct and visualize the bulk structure are Mo1 (0.333, 0.667, 0.368), Mo2 (0.667, 0.333, 0.632), C (0.000, 0.000, 0.500), F1 (0.667, 0.333, 0.184) and F2 (0.333, 0.667, 0.8156). The atomic coordinates (Wyckoff positions) and unit-cell parameters are taken from a previous report (Khazaei *et al.*, 2014 ▸) and were optimized using the GGA–PBE functional with the convergence criterion of 510^−6^ eV per atom. The Mo—C and Mo—F bonds are strong, with mixed covalent, metallic and ionic characteristics. On the other hand, the F—F bonds are weaker and more reactive, allowing a monolayer to be easily exfoliated from the bulk (Zhu *et al.*, 2017 ▸). The structure of the monolayer was constructed in *VESTA* starting from the 111 unit cell of the bulk crystal and making the value of the *c* axis five times greater than that of the bulk, thereby obtaining a 115 supercell. The number of layers was increased by adding a layer into the unit cell while keeping the lattice parameter along the *c* axis fixed. The structure from monolayer to multilayer is optimized every time a layer is added. The optimized unit-cell parameters are shown in Table 1 ▸.

The electronic band structures of bulk and 2D layered Mo_2_CF_2_ were calculated based on density functional theory (DFT) within the generalized gradient approximation (GGA) using the Perdew–Burke–Ernzerhof (PBE) functional. The calculations were implemented in the CASTEP code (Segall *et al.*, 2002 ▸; Accelrys, 2010 ▸) with a sufficiently high plane-wave basis cut-off of 500 eV. In all calculations, a 551 Monkhorst–Pack *k*-point grid was used to generate the initial charge density.

The optical properties of the bulk and 2D layered Mo_2_CF_2_ were extracted from the frequency-dependent com­plex di­elec­tric function after applying a scissors correction to account for the excited-state nature of the optical properties. The com­plex dielectric function represents the linear response of the system to an external electromagnetic field and consists of a real and imaginary part as follows:

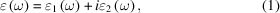

where ω is the optical frequency and ɛ_1_(ω) and ɛ_2_(ω) are the real and imaginary parts of the dielectric function, respectively. The real part ɛ_1_(ω) of the dielectric function can be obtained from the imaginary part ɛ_2_(ω) using the Kramers–Kronig relationships (Hutchings *et al.*, 1992 ▸; Kronig, 1926 ▸; Kramers, 1927 ▸).

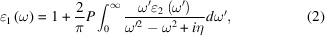

where *P* is the principal value, η is an infinitesimal com­plex shift with a value of 0.1 and ω is the frequency over which the equation is being integrated. From the frequency-dependent com­plex dielectric function, other optical parameters, such as the absorption coefficient α(ω), refractive index *n*(ω), reflectivity *R*(ω) (Qiu *et al.*, 2018 ▸) and conductivity (σ) (Accelrys, 2010 ▸), were calculated using the following equations, where *c* is the speed of light:

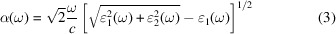




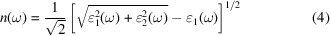




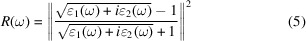




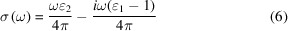




## 3. Results and discussion

### 3.1. Crystal structure and electronic band structure

The unit-cell parameters and bond lengths for the bulk and 2D layered Mo_2_CF_2_ are summarized in Table 1 ▸. Our result for the monolayer is in good agreement with previous reports that numerically calculated the electronic structure of monolayer Mo_2_CF_2_ (Khazaei *et al.*, 2014 ▸). Experimental results for the band gap energy, unit-cell parameters, and bond lengths of bulk and 2D layered Mo_2_CF_2_ are still lacking.

**Figure 2:**
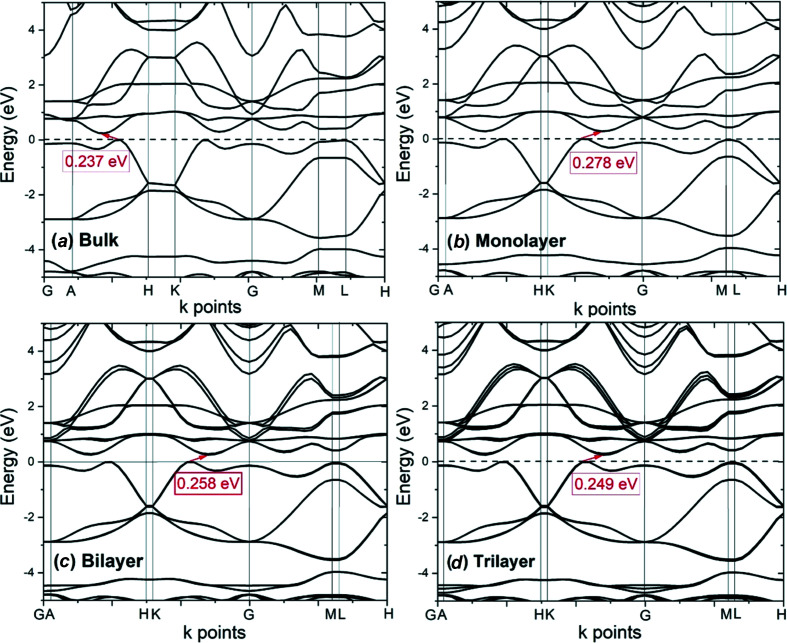
Electronic band structures of (*a*) bulk, (*b*) monolayer, (*c*) bilayer and (*d*) trilayer Mo_2_CF_2_

Figs. 2 ▸(*a*)–2 ▸(*d*) show the electronic band structures of bulk and 2D layered Mo_2_CF_2_ calculated along the high-symmetry *k*-points path *G–A–H–K–G–M–L–H* in the first Brillouin zone. In these figures, the valence band maxima are shifted to zero energy. The band structures exhibit similar band shapes, particularly for the valence and conduction bands. All the materials are seen to be indirect band gap semiconductors. The maximum of the valence band and the minimum of the conduction band of the monolayer and multilayer are located at the *k*-point between *G* and *K*, while the bulk has the band gap located at the *k*-point between *H* and *A*.

The band gap energies of the monolayer, bilayer, trilayer and bulk Mo_2_CF_2_ are 0.278, 0.258, 0.249 and 0.237 eV, respectively, as summarized in Table 1 ▸. The 0.278 eV band gap energy obtained from our calculations for the monolayer is in good agreement with the previously reported band gap energy of 0.27 eV calculated using GGA–PBE (Khazaei *et al.*, 2014 ▸). In general, the band gap energy decreases as the number of layers is increased. The decrease in band gap energy can be attributed to inter­layer coupling, which results in the splitting of the bands. As can be seen in Figs. 2 ▸(*b*) (monolayer), 2 ▸(*c*) (bilayer) and 2 ▸(*d*) (trilayer), band splitting increases progressively from the monolayer to the trilayer, and the splitting depends on the number of layers. As the number of layers increases, the splitting of bands due to the stronger inter­layer coupling results in a decrease in the band gap energy. In com­parison, band splitting in the bulk Mo_2_CF_2_ is relatively small, which indicates that the couplings in the bulk are weak van der Waals inter­actions. These results indicate that the band gap of 2D layered Mo_2_CF_2_ could be engineered by controlling the number of layers. The ability to manipulate the band gap energy makes Mo_2_CF_2_ a promising material for applications in the field of electronic devices.

**Figure 3:**
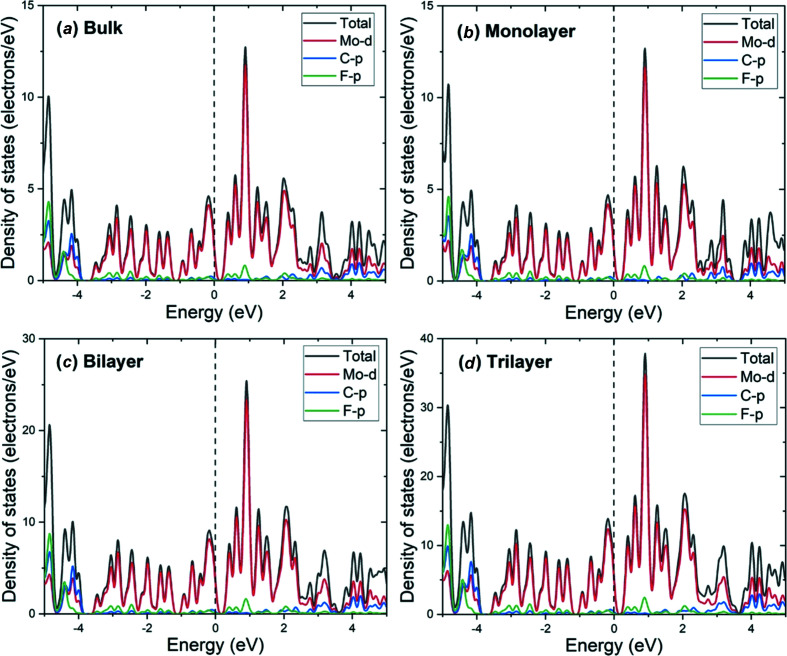
Total and partial density of states for (*a*) bulk, (*b*) monolayer, (*c*) bilayer and (*d*) trilayer Mo_2_CF_2_. The dashed lines represent the Fermi level.

The total and partial density of states for the bulk and 2D layered Mo_2_CF_2_ are shown in Fig. 3 ▸. The valence band maximum and conduction band minimum are mainly com­posed of Mo-*d* states with a small overlap with C-*p* and F-*p* states. The Mo-*d* states also play a dominant role in the conduction band above the Fermi level, which confirms the partially occupied Mo^2+^ 4*d* ^2^ orbital.

### 3.2. Optical properties

**Figure 4:**
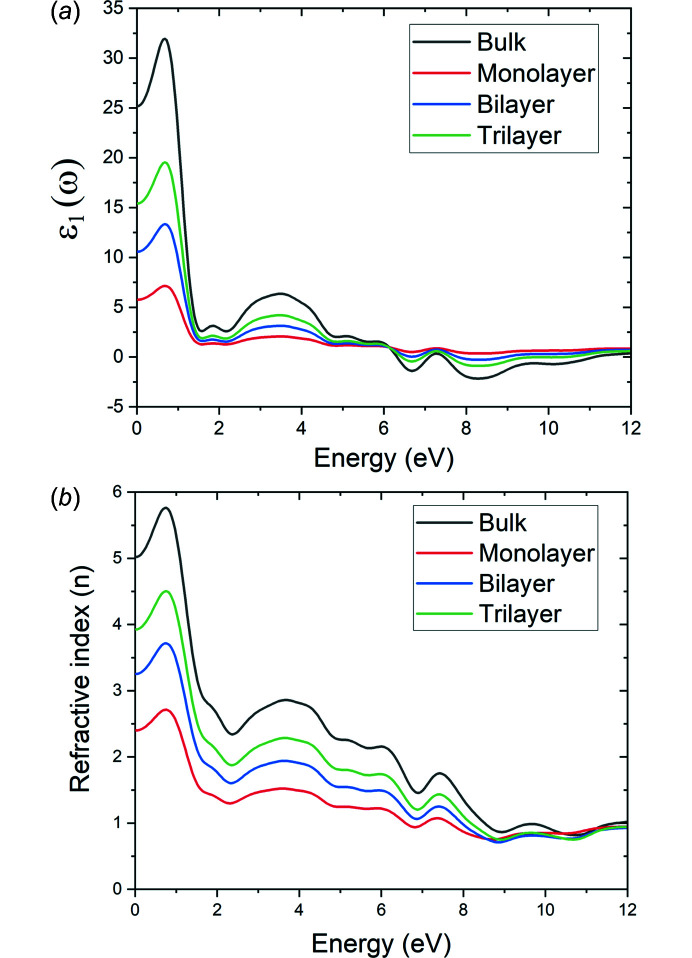
(*a*) Real part of the dielectric function [ɛ_1_(ω)] and (*b*) refractive index (*n*) as a function of incident photon energy for bulk and 2D layered Mo_2_CF_2_.

Fig. 4 ▸(*a*) shows the real part of the dielectric function, ɛ_1_(ω), for the bulk and 2D layered Mo_2_CF_2_. The calculated values of ɛ_1_(0) are 5.76, 10.58 and 1.42 eV for the monolayer, bilayer and trilayer Mo_2_CF_2_, respectively. From 0 to 6 eV, the intensity of the peaks is seen to increase as the number of layers increases. The maximum value for ɛ_1_(ω) is achieved at around 0.67 eV for all the materials. The higher value of ɛ_1_(ω) shows a greater ability for the polarization of low incident photon energy as the number of layers increases. Moving towards higher incident photon energy, an inflection point is observed around 6 eV, where ɛ_1_(ω) takes on negative values. Above 6 eV, the intensity of the peaks appears to decrease (becomes more negative) as the number of layers increases. In the photon energy inter­vals 0.68–2.19, 3.49–6.68 and 7.30–8.31 eV, the bulk and 2D layered Mo_2_CF_2_ behave as a metal owing to anomalous dispersion. Dispersion is the property of materials whereby the refractive index changes as a function of wavelength. In normal dispersion, the refractive index increases as the photon energy increases (wavelength decreases), meaning that longer wavelengths are bent less com­pared to shorter wavelengths. The anomalous dispersion is confirmed by the plot of the refractive index as a function of photon energy [Fig. 4 ▸(*b*)]. Here, the refractive index decreases as the photon energy increases in the photon energy inter­vals 0.76–2.41, 3.66–6.89 and 7.43–8.94 eV. In normal dispersion, the refractive index should increase as the photon energy increases, as observed in the ranges 0–0.76, 2.41–3.66 and 6.89–7.43 eV. It is also inter­esting to note that when the incident photon energy is greater than 8.52 eV, the value of the refractive index is less than one, which is characteristic of metals. Comparing the refractive index values of the 2D layered Mo_2_CF_2_, the refractive index can be increased by increasing the number of layers. These results suggest that the static dielectric constant and the optical dispersion characteristics of the 2D layered Mo_2_CF_2_ can also be manipulated by controlling the number of layers.

**Figure 5:**
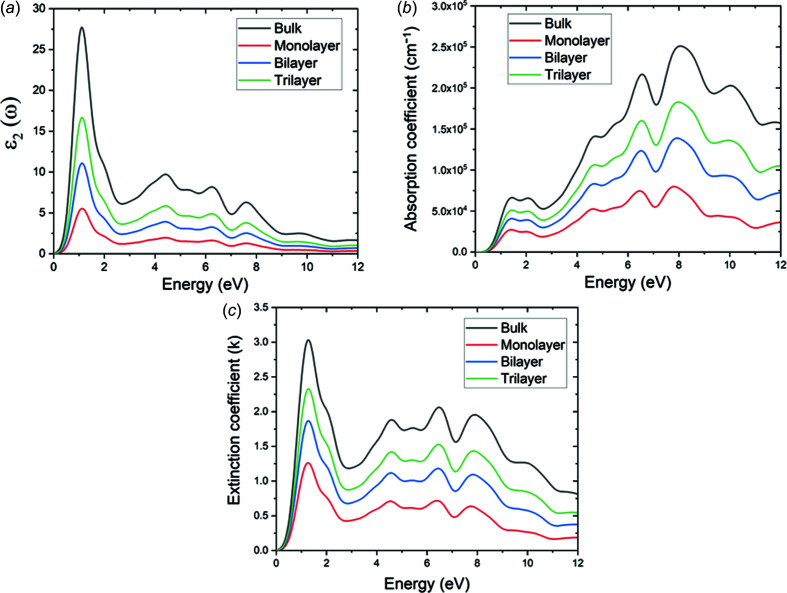
(*a*) Imaginary part of the dielectric function [ɛ_2_(ω)], (*b*) absorption coefficient and (*c*) extinction coefficient (k) as a function of incident photon energy for bulk and 2D layered Mo_2_CF_2_.

The imaginary part of the dielectric function, ɛ_2_(ω), is shown in Fig. 5 ▸(*a*). This com­ponent is related to the transitions between the valence and conduction bands, and therefore to the electronic structure of Mo_2_CF_2_ and the absorption of the incident photons. The optical band gaps of the bulk and 2D layered Mo_2_CF_2_ can then be estimated from ɛ_2_(ω) and these are 0.220, 0.178 and 0.136 eV for the monolayer, bilayer and trilayer Mo_2_CF_2_, respectively. These optical band gap energies are smaller than the electronic band gap energies obtained from the electronic structures (and summarized in Table 1 ▸). The discrepancy comes from the Coulombic inter­action between electrons in the conduction band and holes in the valence band, as well as the exclusion of excitonic effects in the approximation (Cadatal-Raduban *et al.*, 2020 ▸). Nevertheless, a similar trend to the electronic band gap energy is observed, wherein the optical band gap decreased as the number of layers increased. Above the optical band gap energy, a sharp rise in the intensity of ɛ_2_(ω) is observed, with its peak appearing at 1.12 eV for all the materials. The peak corresponds to the electron transitions between the Mo-4*d* states to the Mo-5*d* states, as indicated by the density of states of Mo_2_CF_2_ (Fig. 3 ▸). The absorption coefficient and extinction coefficient of bulk and 2D layered Mo_2_CF_2_ is shown in Figs. 5 ▸(*b*) and 5 ▸(*c*), respectively. Regardless of the number of layers, Mo_2_CF_2_ is seen to be absorbing over a broad range of photon energies up to the vacuum ultraviolet region (greater than 6 eV photon energy or less than 200 nm wavelength), with a lower photon energy cut-off of 0.429, 0.387 and 0.345 eV for the monolayer, bilayer and trilayer nanosheets, respectively, which is in the mid-infrared wavelength region (wavelengths of about 2890, 3204 and 3594 nm for the monolayer, bilayer and trilayer nanosheets, respectively). Its peak absorption range is in the vacuum ultraviolet region from about 6 to 11 eV (wavelengths of about 207 to 112 nm), with an absorption peak at around 7.9 eV (157 nm). The extinction coefficient measures the loss of photon energy due to absorption and scattering, and therefore mimics the trend of ɛ_2_(ω). Like the trend observed for ɛ_2_(ω), where the intensity of ɛ_2_(ω) increased as the number of layers increased, the value of the absorption and extinction coefficients also increased as a function of the number of layers. This indicates that the absorption of Mo_2_CF_2_ can also be manipulated by varying the number of layers.

**Figure 6:**
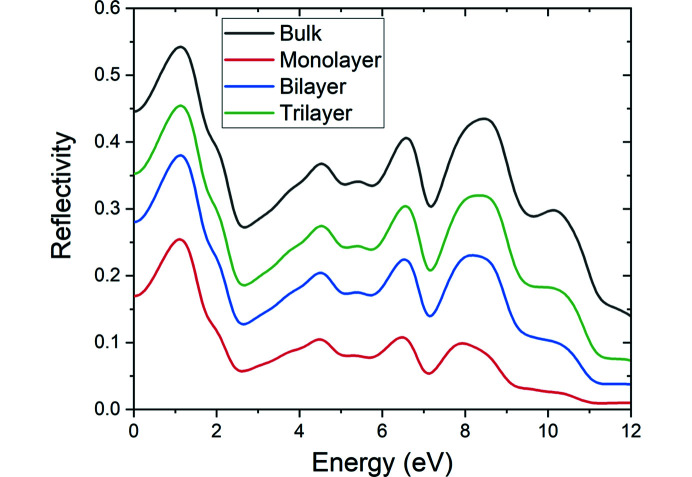
Reflectivity of bulk and 2D layered Mo_2_CF_2_.

Fig. 6 ▸ shows the reflectivity of bulk and 2D layered Mo_2_CF_2_. The reflectivity of the bilayer and trilayer Mo_2_CF_2_ are 0.28 and 0.35 at 0 eV, which means that about 28 and 35% of the incident light is reflected. A relatively low reflectivity is observed as the incident photon energy increases. In general, the reflectivity has a decreasing trend at photon energies between about 5 and 12 eV (about 248 and 103 nm). As reflectivity defines the ability of a material to reflect the incident photons, Mo_2_CF_2_ could be suitable as an antireflective coating, especially in the vacuum ultraviolet wavelength region. Furthermore, for this purpose, a smaller number of layers appears to be more advantageous (for example, a monolayer com­pared to a trilayer) as the general trend is that the reflectivity decreases with a decrease in the number of layers.

**Figure 7:**
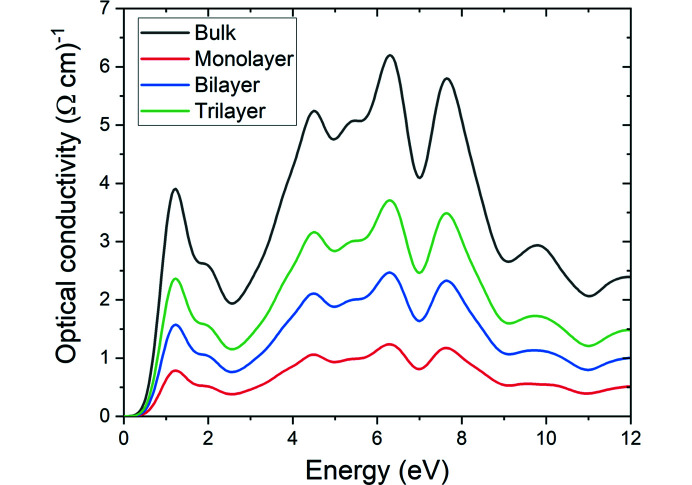
Optical conductivity of bulk and 2D layered Mo_2_CF_2_.

Fig. 7 ▸ shows the optical conductivity of bulk and 2D layered Mo_2_CF_2_. The optical conductivity is derived from ɛ_2_(ω) and therefore displays a similar trend com­pared to the absorption and extinction coefficient spectra [Figs. 5 ▸(*b*) and 5 ▸(*c*)]. Optical conductivity peaks are observed around 0.76–2.41, 3.66–6.89 and 7.43–8.94 eV. The value of ɛ_1_(ω) is negative [Fig. 4 ▸(*a*)] and the materials display anomalous dispersion [Fig. 4 ▸(*b*)] within these energy ranges, affirming the metallic behavior of Mo_2_CF_2_ at these energy ranges.

## 4. Conclusions

Using first principles density functional theory within the generalized gradient approximation (GGA) and the Perdew–Burke–Ernzerhof (PBE) functional, the electronic structure and optical properties of bulk and 2D layered (monolayer, bilayer and trilayer) Mo_2_CF_2_ were studied. The band gap energy of the 2D layered Mo_2_CF_2_ decreased as the number of layers increased due to inter­layer coupling, which resulted in the splitting of the bands. Investigation of the optical properties of Mo_2_CF_2_ revealed that it behaves as a metal with an anomalous dispersion in the photon energy inter­vals of 0.68–2.19, 3.49–6.68 and 7.30–8.31 eV. Mo_2_CF_2_ also exhibited a high optical conductivity within these energy inter­vals. Regardless of the number of layers, Mo_2_CF_2_ was seen to be absorbing over a broad range of photon energies up to the vacuum ultraviolet region (greater than 6 eV photon energy or less than 200 nm wavelength), with a lower photon energy cut-off of 0.429 (2890), 0.387 (3204) and 0.345 (3594 nm) for the monolayer, bilayer and trilayer nanosheets, respectively, which is in the mid-infrared wavelength region. Its peak absorption range is in the vacuum ultraviolet region from about 6 to 11 eV (wavelengths of about 207 to 112 nm). A relatively low reflectivity is observed as the incident photon energy increases, particularly at photon energies between about 5 to 12 eV (about 248 to 103 nm). The band gap energy, static dielectric constant, optical dispersion, absorption, extinction coefficient, reflectivity, and conductivity of the 2D layered Mo_2_CF_2_ can be manipulated by controlling the number of layers. The unique behavior of its optical properties along with the ability to control its electronic and optical properties indicate the huge potential of 2D layered Mo_2_CF_2_ for various applications in the field of electronic devices.

## Figures and Tables

**Table 1:** Calculated band gap energy, unit-cell parameters and bond lengths for the bulk and two-dimensional layered Mo_2_CF_2_

	Band gap energy (eV)	*a* = *b* (Å)	*c* (Å)	*d*_Mo—C_ (Å)	*d*_Mo—F_ (Å)
Bulk	0.237	3.2738	6.7495	2.1125	2.2960
Trilayer	0.249	3.2798	35.7058	2.1154	2.3036
Bilayer	0.258	3.2793	35.7128	2.1151	2.3032
Monolayer	0.278	3.2786	35.7215	2.1148	2.3029
